# Dataset for image classification with knowledge

**DOI:** 10.1016/j.dib.2024.110893

**Published:** 2024-09-05

**Authors:** Franck Anaël Mbiaya, Christel Vrain, Frédéric Ros, Thi-Bich-Hanh Dao, Yves Lucas

**Affiliations:** a University Orleans, INSA Centre Val de Loire, LIFO, EA 4022, France; b University Orleans, INSA Centre Val de Loire, PRISME, EA 4229, France

**Keywords:** Computer vision, Image classification, Knowledge, Deep learning, Rules

## Abstract

Deep learning applied to raw data has demonstrated outstanding image classification performance, mainly when abundant data is available. However, performance significantly degrades when a substantial volume of data is unavailable. Furthermore, deep architectures struggle to achieve satisfactory performance levels when distinguishing between distinct classes, such as fine-grained image classification, is challenging. Utilizing a priori knowledge alongside raw data can enhance image classification in demanding scenarios. Nevertheless, only a limited number of image classification datasets given with a priori knowledge are currently available, thereby restricting research efforts in this field. This paper introduces innovative datasets for the classification problem that integrate a priori knowledge. These datasets are built from existing data typically employed for multilabel multiclass classification or object detection. Frequent closed itemset mining is used to create classes and their corresponding attributes (e.g. the presence of an object in an image) and then to extract a priori knowledge expressed by rules on these attributes. The algorithm for generating rules is described.

Specification Table

**Table:** 

Subject	Computer Vision and Pattern Recognition
Specific subject area	Datasets for image classification are enhanced with a priori knowledge structured as attributes and rules. Our datasets can be used for machine learning and deep learning tasks such as image classification, prediction, and concept embedding. The image attributes represent semantic concepts inherent to the image, which include the objects present in it. The rules are generated from the created classes.
Type of data	The datasets include images in JPG format with annotated labels. The knowledge, formalized as attributes and rules, is contained within a CSV file.
Data collection	Data was collected from MS COCO 2014 dataset [1]. The different classes are created using the ECCLAT method [2], which extracts a subset of concepts from frequent closed objects in the images, using an evaluation measure. This evaluation measures the ranking of frequent closed objects (treated as clusters) based on their internal and external similarities. The different classes are created from this hierarchy, prioritizing the most relevant frequent closed objects containing a minimum number of images. At the end of this process, the objects contained in each image are stored. These objects are then used to create rules to characterize the different classes.
Data source location	The datasets were created from MS COCO 2014 [1].
Data accessibility	Data repository name: Dataset for image classification with knowledge Data identification number: DOI: 10.17632/zr36dyjtjr.1 Direct URL to data: https://data.mendeley.com/datasets/zr36dyjtjr/1

## 1 Value of the Data

• Prior knowledge, especially deep architectures, is becoming increasingly common in research [3]. Deep networks require a large amount of training data. The use of prior knowledge is a common approach to reduce the amount of required training data. In computer vision, datasets containing prior knowledge are scarce. The availability of such data will help researchers develop more innovative machine learning approaches to image classification, object detection, and concept embeddings, using prior knowledge formalized as attributes or rules.
• The computer vision research community can use the proposed datasets for concept embedding or image classification tasks based on images, concepts, and rules. These datasets can serve as a foundation for developing high-performing deep architectures that do not require much training data or contain challenging classification tasks.
• These datasets can be employed in concept-based classification to assess the explainability of models operating within this paradigm.
• The method proposed is generic and can be customized for creating datasets from various sources. Parameters can be adjusted to fit specific requirements.

## 2 Background

Deep learning architectures have proven to be highly effective in computer vision tasks. However, these models typically require a large volume of image data for training. Recent research aims to reduce the amount of data needed by incorporating prior knowledge during the learning process [3]. However, datasets that include such knowledge in computer vision are scarce. Our goal is to provide the community with new datasets that enable researchers to evaluate deep learning models that integrate knowledge. These datasets include knowledge in the form of attributes and rules, offering comprehensive resources for training and validating knowledge-based computer vision models. Moreover, in concept-based explainable classification [4], attributes can be viewed as concepts present in the images. Therefore, our datasets are also suitable for experiments in this context, including rules associated with different classes.

## 3 Data Description

In recent years, there has been a growing trend in machine learning research to leverage prior knowledge in conjunction with raw data to enhance the performance and interpretability of models [3]. However, it remains challenging in computer vision to find publicly available datasets that incorporate prior knowledge. This paper addresses this gap by introducing three distinct datasets: COCO@11, COCO@24, and COCO@48. Each of these datasets is derived from the COCO 2014 dataset [1] through formal concept analysis methods. Notably, each dataset is carefully labeled and serves as a valuable resource for multi-class classification tasks. Furthermore, these datasets are enriched with formalized knowledge in the form of attributes and rules, providing an additional layer of information to complement the raw image data. Each dataset includes both a training set and a test set.

Each dataset is organized into three folders:

• A folder named *images* containing train and test images is organized into classes. The images are all in JPG format, with varying dimensions. Importantly, the image names remain unchanged, aligning with the MS COCO dataset.
• A folder named *attributes* contains two CSV files corresponding to the training and test images. Each row in CSV file corresponds to an image, and each column corresponds to an object (presence or absence in the image) except for the two last columns, which respectively contain the image path and the image class. Boolean values are associated with each image for the columns corresponding to the object, depending on whether the corresponding object is present or not in the image (1 = present, 0 = not present).
• A folder named *rules* contains two text files corresponding to two class rule types. Let us define the absolute support of a rule A→Bin dataset Das the number of images of Dthat satisfy *A*. Let us define the confidence of a rule A→Bin dataset Das the ratio of images of Dthat satisfy Bamong the images that satisfy A. There are two types of class rules:

- the rule $Cl\to A_{1}\wedge A_{2}\wedge \ldots \wedge A_{j}$ has a confidence equal to 1 on the training set. This is no longer true on the test set: some images of the test set of class Clmay not satisfy the rule's conclusion.
- the rules $B_{1}\wedge B_{2}\wedge \ldots \wedge B_{j}\to Cl$ have a support greater than 1 and a confidence equal to 1 in the training set. This means that at least one image of the training set satisfies $B_{1}\wedge B_{2}\wedge \ldots \wedge B_{j}$ and all the images in the training set that satisfying $B_{1}\wedge B_{2}\wedge \ldots \wedge B_{j}$ satisfy Cl. This may no longer hold true for the test set.

In these rules, Clrepresents a class and $A_{i}$*_,_* $B_{j}$ represents the presence of objects in the images. (e.g. $\left(mouse\wedge bowl\wedge tv\right)\to Class_{2}$)

Each class rule file contains two columns delimited by a comma. The first column represents the rule's premise and conclusion, with the two parts separated by the →symbol. The second column indicates for a rule C→Dthe ratio of images satisfying Camong those satisfying Don the training set. It is always equal to 1 for the first kind of rule.

### 3.1 COCO@11

**Fig. 1:**
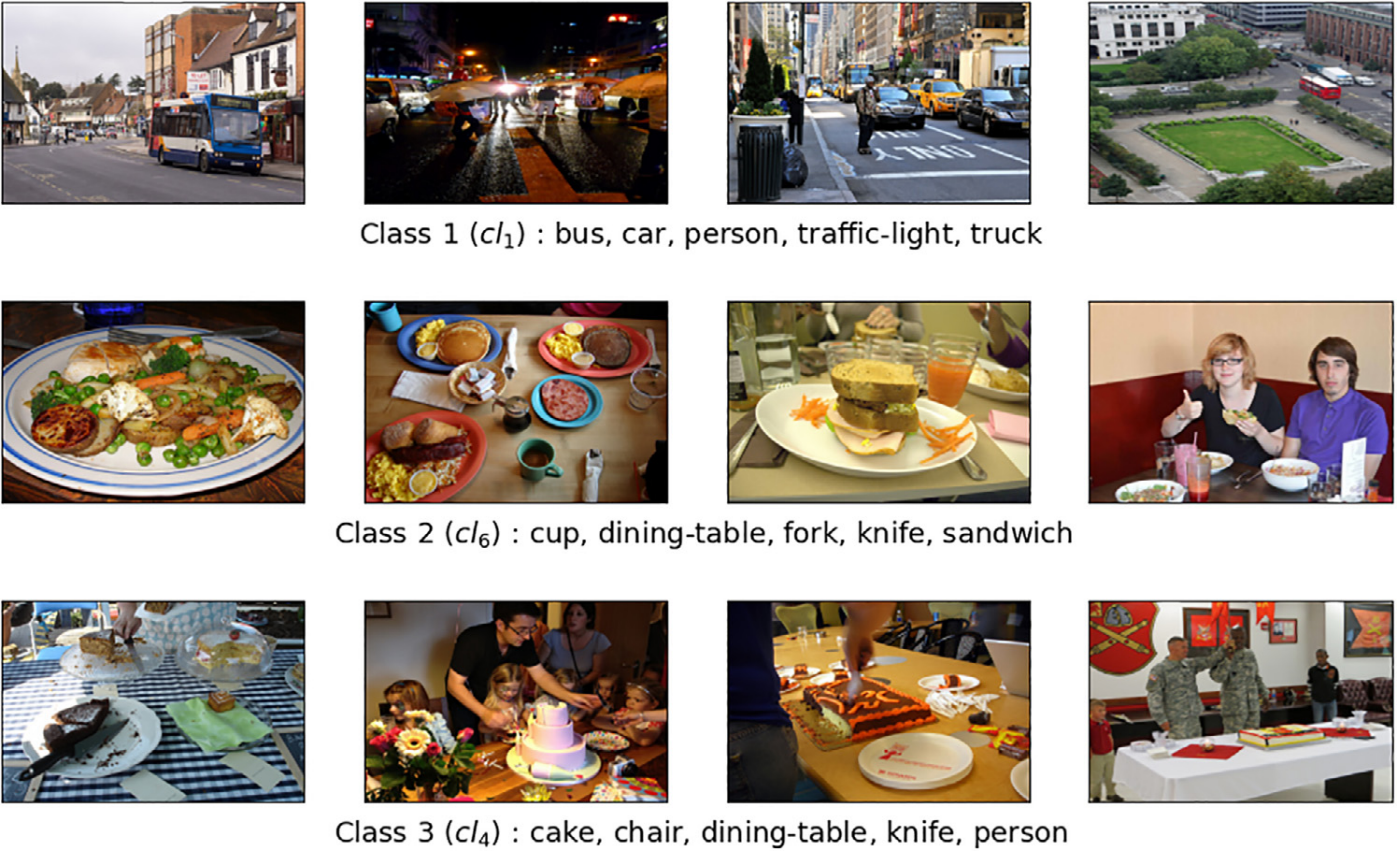
Three classes randomly selected from the COCO@11 dataset.

The dataset COCO@11 contains 11 different classes and 24 distinct objects. It includes a total of 2,463 images, out of which 1,692 are for the training set and 771 are for the testing set. Each class consists of at least 200 images, with at least 5 objects per class, with a total of 24 distinct objects. Table 1 displays the distribution of images across the training and test sets, outlines the defining objects for each class, and provides the total number of class rules generated for each specific class. Fig. 1 illustrates several image examples representing three classes out of the total 11 classes in the COCO@11 dataset. We can see that it is intuitively easier to distinguish the semantics of Class 1 compared to the two other classes. Classes 2 and 3 are semantically closer, but the objects describing them allow them to be distinguished.

**Table 1:** COCO@11 dataset.

Class	Images		Objects describing class	Class Rules
	Train	Test		
*Cl*_1_	141	59	bus, car, person, traffic-light, truck	4
*Cl*_2_	139	61	book, chair, keyboard, mouse, tv	27
*Cl*_3_	134	77	bottle, bowl, cup, oven, sink	53
*Cl*_4_	160	60	cake, chair, dining-table, knife, person	93
*Cl*_5_	143	59	cell-phone, chair, cup, dining-table, person	130
*Cl*_6_	149	71	cup, dining-table, fork, knife, sandwich	53
*Cl*_7_	180	85	chair, cup, dining-table, person, pizza	31
*Cl*_8_	151	61	dining-table, fork, knife, person, pizza	29
*Cl*_9_	173	86	bottle, chair, dining-table, person, wine-glass	43
*Cl*_10_	166	74	bottle, chair, cup, dining-table, person	9
*Cl*_11_	156	78	bowl, cup, dining-table, fork, spoon	15
**Total**	1,692	771	24	487

### 3.2 COCO@24

**Fig. 2:**
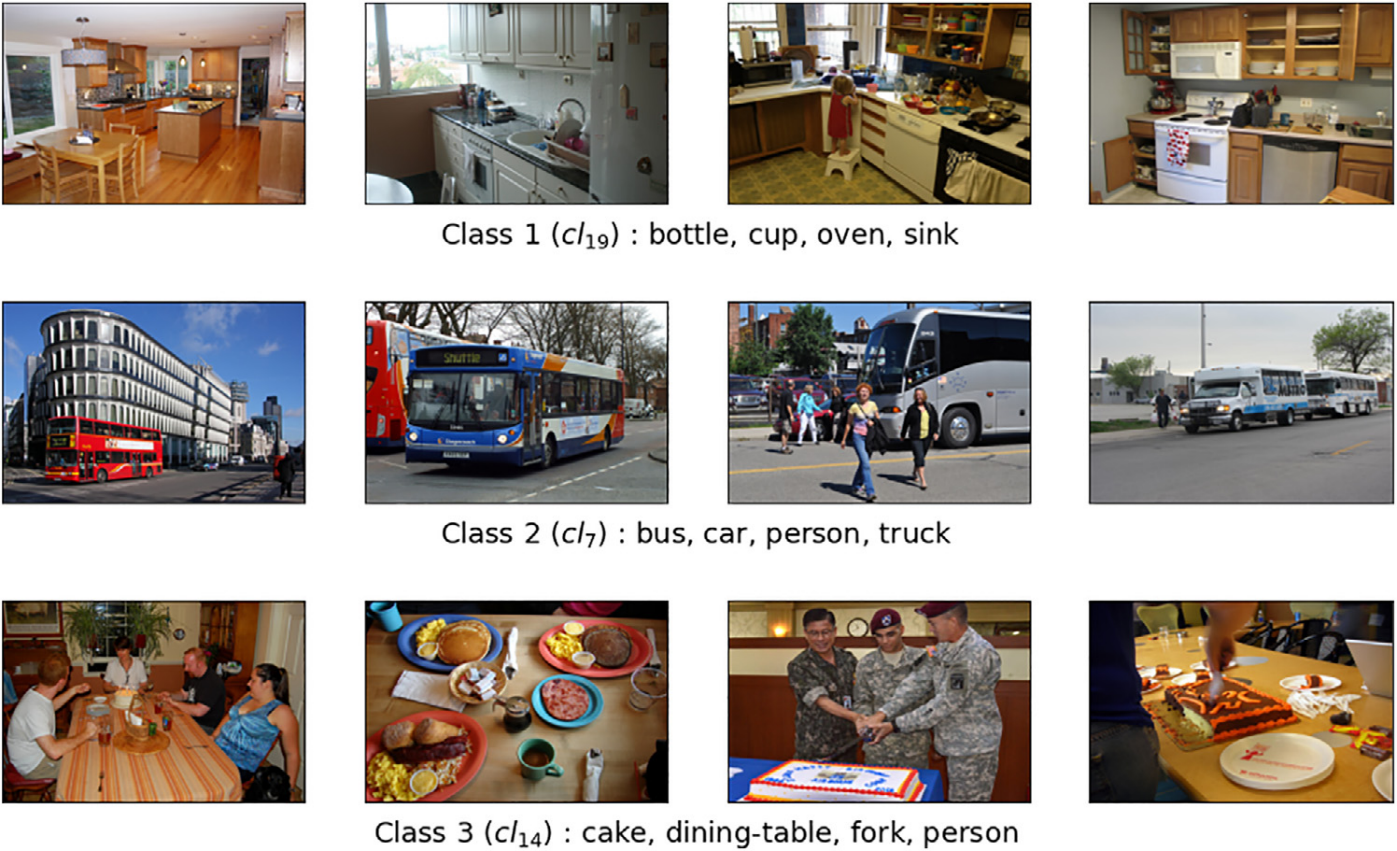
Illustration with three classes randomly selected from the COCO@24 dataset.

The dataset COCO@24 consists of 24 classes and a total of 8,601 images. Among these images, 5,879 are for the training set, and 2,722 are for the test set with a distribution of at least 230 images per class and at most 682 images. Each class is characterized by at least 4 objects, and the dataset has 36 distinct objects. Table 2 illustrates the distribution of images in both the training and test sets, outlines the defining objects for each class, and presents the number of generated class rules for each respective class. Fig. 2 presents several image examples representing three classes from the 24 total classes in the COCO@24 dataset. As with the previous dataset, some classes are semantically closer than others. In Fig. 2, Class 2 is clearly distinguished from the other classes. Although Classes 1 and 3 are semantically close, the are link with food or cooking, the objects that describe them show their difference.

**Table 2:** COCO@24 dataset.

Class	Images		Objects describing class	Class Rules
	Train	Test		
*Cl*_1_	241	150	chair, person, sports-ball, tennis-racket	41
*Cl*_2_	470	212	baseball-bat, baseball-glove, person, sports-ball	30
*Cl*_3_	225	121	backpack, handbag, person, suitcase	90
*Cl*_4_	176	79	backpack, handbag, person, umbrella	90
*Cl*_5_	280	142	car, motorcycle, person, truck	46
*Cl*_6_	373	168	car, person, traffic light, truck	37
*Cl*_7_	163	90	bus, car, person, truck	19
*Cl*_8_	285	116	bus, car, person, traffic-light	41
*Cl*_9_	156	82	chair, dining-table, person, umbrella	140
*Cl*_10_	307	94	chair, couch, person, remote	392
*Cl*_11_	222	92	microwave, oven, refrigerator, sink	504
*Cl*_12_	235	109	book, chair, couch, tv	605
*Cl*_13_	163	81	chair, couch, potted-plant, vase	294
*Cl*_14_	221	87	cake, dining-table, fork, person	170
*Cl*_15_	244	101	cake, chair, dining-table, person	249
*Cl*_16_	216	118	cup, dining-table, person, sandwich	242
*Cl*_17_	215	91	cell-phone, cup, dining-table, person	269
*Cl*_18_	304	153	chair, dining-table, person, pizza	111
*Cl*_19_	143	90	bottle, cup, oven, sink	243
*Cl*_20_	194	89	dining-table, fork, knife, pizza	32
*Cl*_21_	161	69	chair, dining-table, person, potted-plant	264
*Cl*_22_	376	178	bowl, cup, dining-table, spoon	204
*Cl*_23_	315	142	chair, cup, dining-table, person	91
*Cl*_24_	194	68	cup, dining-table, fork, knife	35
**Total**	5,879	2,722	24	4,239

### 3.3 COCO@48

**Fig. 3:**
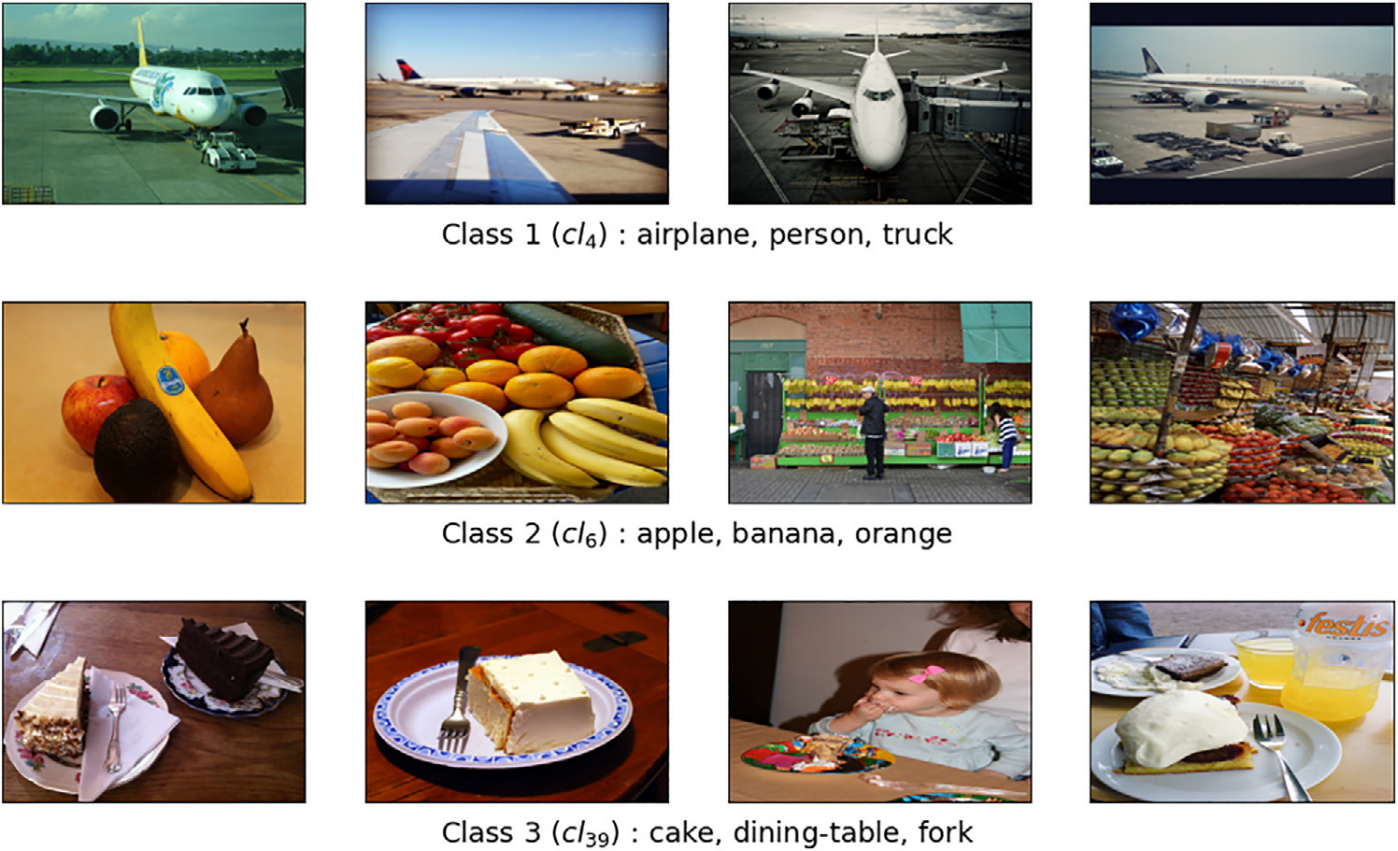
Illustration with three classes randomly selected from the COCO@48 dataset.

The COCO@48 dataset comprises 48 classes and a total of 25,468 images. The training set has 17,278 images, while the test set has 8,190 images. Each class has a minimum of 300 images and a maximum of 1,314 images (training and test set). Additionally, each class is described by at least 3 objects, and there are a total of 59 distinct objects in the dataset. Table 3 shows the distribution of images of the training and test sets, the characteristic objects for each class, and provides the number of generated class rules for each specific class. Fig. 3 illustrates several image examples representing three classes from the total 48 classes in the COCO@48 dataset. As in the two previous datasets, some classes are semantically closer than others.

**Table 3:** COCO@48 dataset.

Class	Images		Objects describing class	Class Rules
	Train	Test		
*Cl*_1_	448	226	backpack, person, skis	29
*Cl*_2_	260	109	person, skis, snowboard	10
*Cl*_3_	294	155	bottle, sink, toilet	41
*Cl*_4_	253	115	airplane, person, truck	34
*Cl*_5_	219	120	bench, person, skateboard	34
*Cl*_6_	211	89	apple, banana, orange	710
*Cl*_7_	381	180	car, person, skateboard	99
*Cl*_8_	815	360	baseball glove, person, sports ball	69
*Cl*_9_	228	118	car, horse, person	103
*Cl*_10_	250	115	handbag, person, train	104
*Cl*_11_	452	267	chair, person, tennis racket	87
*Cl*_12_	208	112	bench, person, umbrella	299
*Cl*_13_	263	122	car, person, stop sign	187
*Cl*_14_	307	135	backpack, person, umbrella	330
*Cl*_15_	749	366	car, motorcycle, person	330
*Cl*_16_	207	118	car, clock, person	192
*Cl*_17_	541	228	cell phone, handbag, person	1,240
*Cl*_18_	222	95	car, fire hydrant, person	55
*Cl*_19_	244	98	baseball bat, bench, person	49
*Cl*_20_	309	152	bench, handbag, person	327
*Cl*_21_	286	157	handbag, person, suitcase	268
*Cl*_22_	344	183	chair, person, umbrella	288
*Cl*_23_	971	449	car, person, truck	281
*Cl*_24_	612	277	bus, car, person	29
*Cl*_2_5	621	355	keyboard, mouse, tv	655
*Cl*_2_6	196	106	car, traffic light, truck	23
*Cl*_2_7	259	127	bowl, broccoli, dining table	520
*Cl*_2_8	348	182	chair, person, tie	389
*Cl*_2_9	245	107	backpack, bicycle, person	116
*Cl*_3_0	273	124	car, person, traffic light	34
*Cl*_3_1	214	95	bottle, person, remote	1,450
*Cl*_3_2	380	182	book, couch, tv	1,363
*Cl*_3_3	353	143	cake, dining table, knife, person	788
*Cl*_34_	390	147	chair, person, remote	416
*Cl*_35_	337	132	microwave, oven, refrigerator	2,492
*Cl*_36_	396	198	chair, laptop, person	1,304
*Cl*_37_	187	114	bottle, cell phone, person	373
*Cl*_38_	221	103	couch, potted plant, vase	542
*Cl*_39_	258	106	cake, dining table, fork	142
*Cl*_40_	522	262	cup, dining table, pizza	246
*Cl*_41_	453	215	cup, dining table, sandwich	407
*Cl*_42_	187	114	book, chair, person	468
*Cl*_43_	323	147	chair, dining table, vase	805
*Cl*_44_	245	102	bowl, oven, spoon	644
*Cl*_45_	248	111	bottle, bowl, sink	688
*Cl*_46_	908	406	chair, dining table, person	568
*Cl*_47_	353	147	bowl, dining table, spoon	373
*Cl*_48_	287	119	dining table, fork, knife	156
**Total**	17,278	8,190	48	20,158

Table 4 presents the image classification performance using classic Convolutional Neural Network methods on each created dataset. The classification task across these diverse datasets is recognized as challenging, highlighting the potential advantages of incorporating knowledge.

**Table 4:** Image classification accuracy using classic Convolutional Neural Network methods on different datasets.

Architecture	Accuracy (%)		
	COCO@11	COCO@24	COCO@48
VGG-19 [5]	51.9	50.1	47.7
ResNet-18 [6]	55.8	55.2	52.8
ResNet-50 [6]	61.0	57.8	56.4
ResNet-101 [6]	59.8	58.7	56.7
ViT-B-16 [7]	65.9	63.9	59.7

The different class rules were generated based on classes, which were created from training images. The second column in Table 5 represents the number of rules generated from training images, while the third column represents the number of rules that are not true in the test set.

**Table 5:** Number of rules that are not true in the test set of each dataset.

Dataset	N. of rules	N. false rules in test
COCO@11	487	157
COCO@24	4,239	1,387
COCO@48	20,158	6,662

## 4 Experimental Design, Materials, and Methods

The COCO@x datasets, where x indicates the number of classes, originate from the initial release (2014) of MS COCO (Microsoft Common Objects in Context) [1]. This dataset is a comprehensive collection for object detection, consisting of approximately 82,000 training images, 41,000 validation images, and 41,000 testing images. Classes do not label images. It encompasses 80 distinct objects distributed throughout the images, with an average of 2.9 objects per image in the training set. Only the training and the validation sets are used since the test set does not contain information on the presence of objects in images. The validation set is used to build our test set.

The datasets introduced in this paper are constructed from MS COCO, taking advantage of the distribution of diverse objects within the images. Each COCO@x dataset serves the purpose of an image classification task. The various classes formed for each dataset are established using formal concept analysis theory applied to the presence of 80 objects, considered as attributes.

**Fig. 4:**
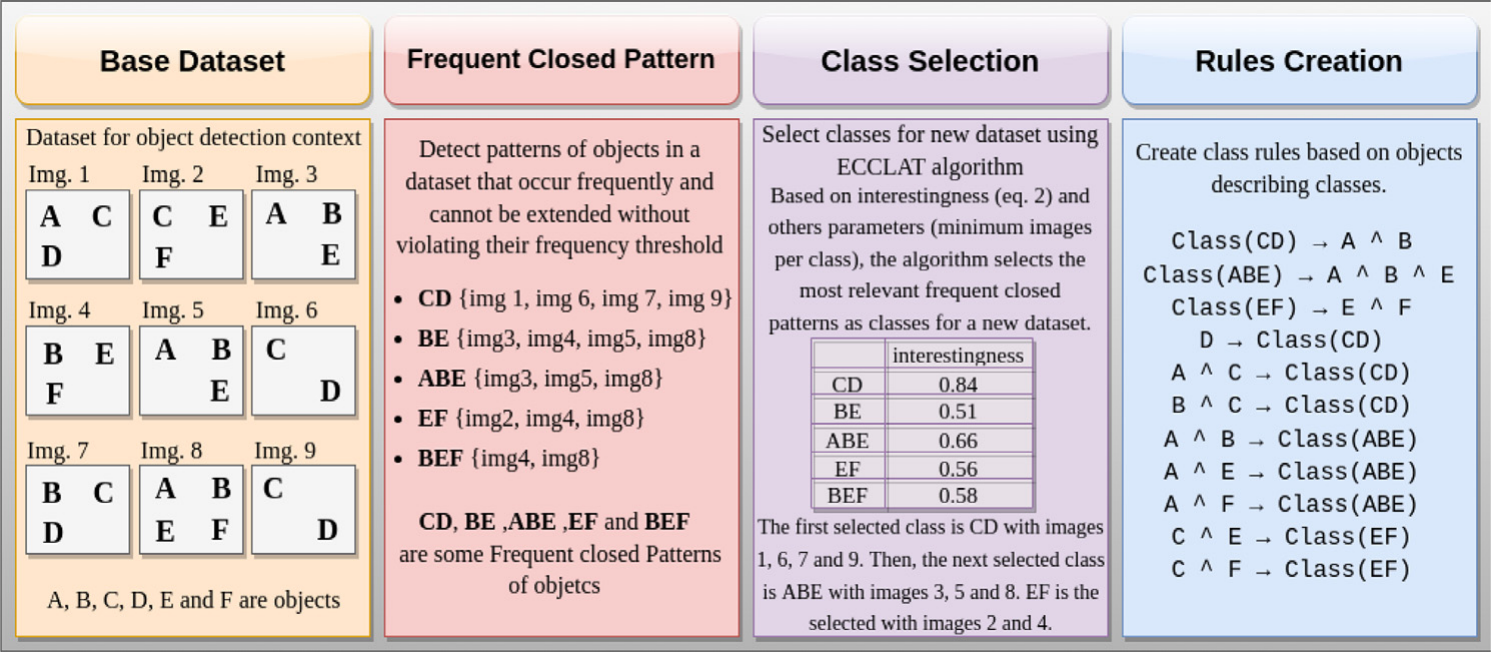
Steps to create each dataset.

The production of COCO@x datasets from MS COCO involves three main stages (Fig. 4). The process begins by generating frequent closed itemsets from the objects depicted in the images. In the second step, classes are defined utilizing the ECCLAT [2] algorithm. The last step involves creating knowledge formalized by rules derived from the classes and the objects describing them.

The creation of classes is based on formal concept analysis. Each class is associated with a closed pattern, a set of Boolean descriptors (indicating the presence of an object in the image). When a pattern is closed, its properties are satisfied only by the objects in the class and are not satisfied by objects in another class. This allows us to characterize the properties of a class. When applying the ECCLAT method to build classes, the condition of having a closed pattern for each class is relaxed to ensure to get a partition.

### 4.1 Formal Concept Analysis

Formal Concept Analysis [8] is a method for analyzing and structuring concepts. Let D=T,I,R) be a transactional dataset where T is a set of transactions, I is a set of items, and R ⊆ T × I is a binary relation between transactions and items. Each couple (*t, i*) ∈ R denotes the fact that the transaction *t* is related to the item *i*. For *T* ⊆ T and *I* ⊆ I, the operators *f* and *g* are defined as:

f(T)={i∈I|∀t∈T,(t,i)∈R}

g(I)={t∈T|∀i∈I,(t,i)∈R}

The set *f*(*T*) is the collection of items shared by all transactions in *T*, and the set *g*(*I*) is the collection of transactions related to all items in *I*. A concept is a pair (*T, I*) where *T* ⊆ T and *I* ⊆ I satisfying *f*(*T*) = *I* and *g*(*I*) = *T*. An itemset is a subset of I. A transaction *t* supports an itemset *I* if *t* ∈ *g*(*I*).

In our context, we are working with a dataset of images containing objects. Consequently, transactions will be denoted as images, and items will be referred to as objects. The pair (*t, i*) ∈ R then represents the presence of object *i* in image *t*. An itemset will be termed as a pattern of objects. We use frequent closed patterns of objects to quantify the similarity between images based on their contained objects, as defined in the following section.

### 4.2 Frequent Closed Pattern of Objects

A closed pattern of objects is a maximal set of objects shared by a set of images, and only by them. More formally, *X* is a frequent closed pattern of objects if *f ° g*(*X*) = *X*. Table 6 illustrates an object recognition context containing 9 images, each identified by its unique ID, and 6 objects labeled from A to F. A cross within the table means the existence of an object in an image. This table serves as the foundation for the examples presented throughout the remainder of the paper.

**Table 6:** Objects recognition context database.

Images	Objects					
	*A*	*B*	*C*	*D*	*E*	*F*
1	×		×	×		
2			×		×	×
3	×	×			×	
4		×			×	×
5	×	×			×	
6			×	×		
7		×	×	×		
8	×	×			×	×
9			×	×		

We consider closed patterns of objects *X* that satisfy two conditions. First, they must be frequent, i.e. their frequency |*g*(*X*)| (number of images) must be greater than or equal to a specified frequency threshold *minfr* (|*g*(*X*)| ≥ *minfr*). Second, *X* must contain at least *minobj* objects (|*X*| ≥ *minobj*). Each closed pattern is associated with a set of images, that is the set of images that contains the objects of the pattern. Such a set of images will form a candidate class. Considering the basic transactional database example from Table 6, the frequent closed patterns of objects with parameters *minfr* = 2 and *minobj* = 2 are presented in the first column of Table 7. Using the MS COCO dataset, we exclusively used the training set to generate the frequent closed patterns of objects.

**Table 7:** Interestingness of frequent closed patterns of objects (FCP).

FCP	hom.	conc.	inter.
**CD** {1, 6, 7, 9}	$\frac{2\times 4}{2\times 4+(1+0+1+0)}=0.8$	$\frac{1}{4}\times \left(\frac{1}{1}+\frac{1}{1}+\frac{1}{1}+\frac{1}{1}\right)=0.88$	0.84
**BE** {3, 4, 5, 8}	$\frac{2\times 4}{2\times 4+(1+1+1+2)}=0.62$	$\frac{1}{4}\times \left(\frac{1}{2}+\frac{1}{3}+\frac{1}{2}+\frac{1}{4}\right)=0.4$	0.51
**ABE** {3, 5, 8}	$\frac{3\times 3}{3\times 3+(0+0+1)}=0.9$	$\frac{1}{3}\times \left(\frac{1}{2}+\frac{1}{2}+\frac{1}{4}\right)=0.42$	0.66
**EF** {2, 4, 8}	$\frac{2\times 3}{2\times 3+(1+1+2)}=0.6$	$\frac{1}{3}\times \left(\frac{1}{1}+\frac{1}{3}+\frac{1}{4}\right)=0.53$	0.56
**BEF** {4, 8}	$\frac{3\times 2}{3\times 2+(0+1)}=0.86$	$\frac{1}{2}\times \left(\frac{1}{3}+\frac{1}{4}\right)=0.29$	0.58

### 4.3 Class Selection

In the context of the following discussion, the idea is to choose interesting patterns that will form the classes. We use the ECCLAT [2] that uses interestingness to evaluate the relevance of each frequent closed pattern of objects to be a class. Considering the dataset from Tables 6 to 7 gives the interestingness of each frequent closed pattern of objects.

We use the interestingness measure to choose classes from the frequent closed patterns of objects. The algorithm depends on the parameter *min_img*, which corresponds to the minimum number of images not yet classified that a newly selected class must classify. Following the recommendation of [2], we used a value of *min_img* close to *minfr*. Based on the interestingness of each potential class, the class selection algorithm is the following: First, the potential class having the highest interestingness is selected as a class. Then, as long as there are images to classify and some potential classes are left, we select the class having the highest interestingness and containing at least *min_img* images not yet classified.

When considering *min_img* = 2 in the context of the dataset given in Table 7, potential classes are CD, BE, ABE, EF, and BEF. With an interestingness value of 0.84, the initially selected class is CD, comprising images 1, 6, 7, and 9. Following that, the ABE class with interestingness value of 0.66 is chosen with images 3, 5, and 8. Subsequently, images 3, 5, and 9 are removed from the BE, EF, and BEF. EF is then selected, containing images 2 and 4. The frequent closed pattern BEF was excluded as it does not satisfy the minimum image requirement (*min_img*) after the removal of image 8, which is already included in class ABE. Similarly, BE cannot be designated as a class for the same reason.

As shown by the example, some patterns may cover the same images, thus leading to overlapping classes. This explains why given a closed pattern *X*, the ECCLAT algorithm removes the images of *g*(*X*) that have already been put in other classes. This also explains why the rules X→Class are not satisfied on the training dataset, and in Section 4.4 we propose an algorithm to search for rules $B_{1}\wedge B_{2}\wedge \cdots \wedge B_{j}\to \mathrm{Cl}$ that are satisfied on the training dataset.

#### 4.3.1 Details on Creating Datasets

Table 8 shows the parameters used to create the different datasets COCO@x. For the COCO@11 dataset, we used *minfr* = 200 and *minobj* = 5. We obtained 133 frequent closed patterns of objects representing potential classes. Using the class selection algorithm with *min_img* = 200, we obtained 11 classes and 24 objects that describe them. For the COCO@24 dataset, we used the parameters *minfr* = 250 and *minobj* = 4. We obtained 280 frequent closed patterns of objects. With *min_img* = 220, we obtained 24 classes described by 24 distinct objects. For the COCO@48 dataset, we used the parameters *minfr* = 300 and *minobj* = 3. We obtained 443 frequent closed patterns of objects. With *min_img* = 300, we obtained 48 classes described by 48 distinct objects. The objects are those that occur in a pattern defining a class.

**Table 8:** Details on creating COCO@x datasets.

Dataset	*minfr*	*minobj*	FCP	*min_img*	Class	Objects
COCO@11	200	5	133	200	11	24
COCO@24	250	4	280	230	24	24
COCO@48	300	3	443	300	48	48

We generated distinct training and testing sets for each dataset. The training samples for the new datasets were derived from the training images of the COCO 2014 dataset. The validation images from COCO 2014 were employed to constitute the test dataset. The test set was not utilized due to the unavailability of information regarding the presence of objects in the images.

### 4.4 Details on Creating Rules

Let $C={\left\{\mathrm{NameC}l_{i},Cl_{i},O_{i}\right\}}_{i=1}^{k}$ be a newly created dataset containing kclasses, where $NameCl_{i}$ represents the name of the *i*−*th* class, $Cl_{i}$ represents the set of images from the *i*−*th* class, and $O_{i}$ represents a set of objects describing the *i*−*th* class. Note that $\forall i,Cl_{i}\neq \emptyset$, $\forall i,j,i\;\neq j,Cl_{i}\;\cap \;Cl_{j}\;=\;\emptyset \;$and ${⋃}_{i=1}^{k}O_{i}=P$ where P is a set of objects describing the dataset.

In the following, for sake of simplicity, when writing rules on images, $Cl_{i}$ represents the Boolean proposition expressing that an image belongs to the class *Cl*_i_ and $Q_{j}$ or $B_{j}\;$represents the Boolean proposition that the corresponding object belongs to the image.

For each class $Cl_{i}$, let $Q\;=\;f(Cl_{i})$ be the set of objects shared by all the images of class i. By the way, classes are built, and the following rule is defined:

$$Cl_{i}\to \wedge_{j=1}^{\left|Q\right|}Q_{j}$$

has confidence equal to 1.

Let us notice that the rule $\wedge_{j=1}^{\left|Q\right|}Q_{j}\to Cl_{i}$ has not a confidence equal to 1, since some images from one class may match the description of other classes. For example, Image 8 is labeled by the ABE class in Table 7, but it also satisfies the properties of EF. In such a scenario, creating equivalence rules between a class and the conjunction of objects shared by all images in this class is not feasible. Therefore, we propose an algorithm to build rules for each class in the following form:

$$\wedge_{j=1}^{\left|B\right|}B_{j}\to Cl_{i,}\;B\subseteq P$$

where *P* is the set of objects occurring in the dataset, with a support greater than 1 on the class and a confidence equal to 1. Moreover, we look for more general rules (the smaller number of conditions in the condition of the rule) satisfying these conditions. Let us recall that each element *B_i_* on the left-hand side of the rule corresponds to an object and means the presence of this object in the image and that a pattern is a conjunction of objects.

To do so, we associate to each pattern two measures: the number (pos, +) of positive examples (images of class *Cl*) that satisfy the pattern, the number (neg, -) of negative examples (images of a class different from *Cl*) that satisfy the pattern. We build a set enumeration tree, starting from patterns of size 1 and pruning the search tree along two conditions: when *pos* = 0 or when *neg* = 0.

Objects are ordered based on the number of positive examples they cover in the class. Given a pattern *p, pos* = 0 means that no positive examples satisfy it and that it is not possible to build a rule satisfying our conditions with this pattern and with patterns that contain it. Thus, the search tree can be pruned at this node. On the other hand, *neg* = 0 and *pos* ≠ 0 means that the rule p→Cl satisfies the two conditions. Moreover, since all patterns $p^{\text{'}}$ that are more specific to p (p ⊆ p*’)* would lead to more specific rules, the search tree can be pruned at this node. When *pos* ≠ 0 and *neg* ≠ 0, the search continues. Image 5 illustrates the enumeration tree for generating rules for the CD class. Green patterns represent those that yield to a rule, while red patterns denote those that do not result in the creation of a rule.

Algorithm 1 describes the generation of such rules. We have *n* images $I=\{I_{1},\;...,I_{n}\}$, *P* objects $O=\{O_{1},...,O_{p}\}$, and *k* classes $C\;=\;\{Cl_{1},\;...,Cl_{k}\}$. *C* forms a partition of the image set (for all$i,\;Cl_{i}\;\neq \emptyset ,\;I\;=\;\cup Cl_{i},$ for all i,jwith $i\;\neq j,\;Cl_{i}\;\cap \;Cl_{j}=\emptyset$). Each image includes a set of objects and the relationship between images and objects is described using the MaskOmatrix ($MaskO={\left\{v_{ij}\right\}}^{p\times n}$, where $v_{ij}=1\left[O_{i}\in I_{j}\right]$).

**Table:** **Algorithm 1** Rules generation algorithm

**Fig. 5:**
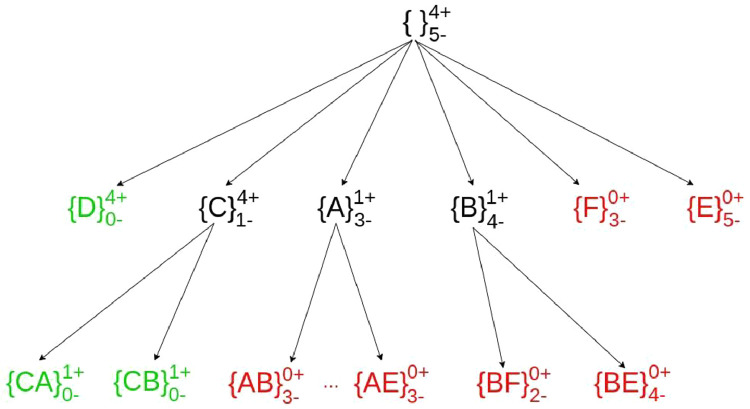
Enumeration tree for generating rules for the class CD.

The way this is implemented is given in Algorithm 1. $Mask^{c}$ and $L_{t}$ are first generated (lines 4,5). $Mask^{c}$ $\left(\in {\left\{0,1\right\}}^{n}\right)$ is a binary vector indicating the images belonging to this class (1 when the image belongs to the class, 0 otherwise) (line 4). $L_{t}$ is a list initialized with patterns of size 1 and updated at each iteration (line 17), to traverse the enumeration tree (Fig. 5).

When the number of positive examples is 0 (line 10), the active pattern does not cover positive examples and cannot be used for building a rule for this class. Then, this pattern is removed from the list $L_{t}$. The number of positive instances is computed by taking the Hadamard product (elementwise multiplication ⊙) between the mask of each object in the active pattern and the mask representing the active class. Let us recall that a pattern is a list of objects, MaskO[p[i]] $\left(\in {\left\{0,1\right\}}^{n}\right)$ is a binary vector indicating whether an image contains the *i*−*th* object of pattern *p*. The number of 1 in the resulting vector gives the number of positive examples. If the number of negative examples is 0 (line 12), it means that the active pattern is satisfied only by images of this class. Then, a novel rule can be generated (line 13). The number of negative examples is calculated similarly to the number of positive examples, but by considering the inverse mask (line 9) of the active class. The algorithm stops when there are no items left to inspect. The class rules derived from the dataset generated based on Table 7 are as follows:

Cl(CD)→(C∧D)

Cl(ABE)→(A∧B∧E)

Cl(EF)→(E∧F)

D→Cl(CD)

(A∧C)→Cl(CD)

(B∧C)→Cl(CD)

(A∧B)→Cl(ABE)

(A∧E)→Cl(ABE)

(A∧F)→Cl(ABE)

(C∧E)→Cl(EF)

(C∧F)→Cl(EF)

## Limitations

The classes are meaningful since they are characterized by a set of properties. However, we acknowledge that the semantics of the classes can still be debatable as the closed pattern condition is further relaxed.

The semantics associated with classes may seem basic. In practice, however, it improves object classification, because if a class is suspected, the probability of finding objects belonging to this class in the image is increased, whereas it is reduced for objects out of context. The greater the number of images containing the same group of objects, the more likely it is that these images accurately describe a real-life situation. The classes are created automatically based on statistical criteria, rather than on the reasoning of a human operator.

## Ethics Statements

Authors affirm that they adhere to ethical guidelines for publishing. Proposed data does not involve any human subjects, animal experiments, or data collected from social media platforms.

## CRediT authorship contribution statement

**Franck Anaël Mbiaya:** Methodology, Conceptualization, Software, Validation, Visualization. **Christel Vrain:** Methodology, Conceptualization, Validation, Supervision. **Frédéric Ros:** Methodology, Supervision, Validation. **Thi-Bich-Hanh Dao:** Methodology, Conceptualization, Validation, Supervision. **Yves Lucas:** Supervision, Validation.

## Data Availability

COCO@x datasets for image classification with knowledge (Original data) (Mendeley Data).
